# Anti-microfouling Activity of *Glycomyces sediminimaris* UTMC 2460 on Dominant Fouling Bacteria of Iran Marine Habitats

**DOI:** 10.3389/fmicb.2018.03148

**Published:** 2019-01-09

**Authors:** Sheida Heidarian, Fatemeh Mohammadipanah, Abdolvahab Maghsoudlou, Yousef Dashti, Gregory L. Challis

**Affiliations:** ^1^Department of Microbiology, School of Biology and Center of Excellence in Phylogeny of Living Organisms, College of Science, University of Tehran, Tehran, Iran; ^2^Ocean Science Research Center, Iranian National Institute for Oceanography and Atmospheric Science, Tehran, Iran; ^3^Department of Chemistry, University of Warwick, Coventry, United Kingdom; ^4^Warwick Integrative Synthetic Biology Centre, University of Warwick, Coventry, United Kingdom; ^5^Department of Biochemistry and Molecular Biology, Monash University, Clayton, VIC, Australia

**Keywords:** surface microlayer, antifouling substances, fouling organisms, marine Actinobacteria, secondary metabolites, marine sediment

## Abstract

Discovery of environmentally safe anti-fouling agent is currently of considerable interest, due to the continuous impact of biofoulers on the marine habitats and the adverse effects of biocides on the environment. This study reports the anti-adhesion effect of marine living Actinobacteria against fouling strains isolated from submerged panels in marine environments of Iran. The extract of *Glycomyces sediminimaris* UTMC 2460 affected the biofilm formation of *Kocuria* sp. and *Mesorhizobium* sp., as the dominant fouling agents in this ecosystem, up to 93.2% and 71.4%, respectively. The metabolic activity of the fouler bacteria was reduced by the extract up to 17 and 9%, respectively. This indicated the bactericidal potency of the extract on cells in the biofilm state that enables the compound to be effective even once the biofilms are established in addition to the inhibition of biofilm initiation. Moreover, extra polymeric substance (EPS) production by fouling bacteria was reduced by 60–70%. The absence of activities against fouling bacteria in suspension and also the absence of toxic effect on *Artemia salina* showed the harmless ecological effect of the anti-microfouling extract on the prokaryotic and eukaryotic microflora of the studied Iran marine ecosystem. Metabolic profiling of *G. sediminimaris* UTMC 2460 revealed the presence of compounds with molecular formulae matching those of known anti-fouling diketopiperazines as major components of the extract. These results suggest that the extract of *Glycomyces sediminimaris* UTMC 2460 could be used as a potentially eco-friendly viable candidate in comparison to the synthetic common commercial anti-microfouling material to prevent the fouling process in marine habitats of Iran.

## Introduction

Colonization and successive overgrowth of epibiotic organisms on submerged surfaces is a natural process, known as biofouling. It causes ecological and economic problems for water-immersed man-made structures and marine sectors such as the loss of productivity in aquaculture, toxicity and side effects of chemical anti-fouling treatments on non-target organisms (Thomas and Brooks, 2010), increased fuel costs for shipping, corrosion of metal pipes, ship and filtration system blockage; an increase in the weight of off-shore sea platforms, in addition to the costs associated with ongoing prevention and control (Garrett et al., 2008). Current anti-fouling strategies inhibit or eradicate the fouling organisms, by releasing high amounts of toxic chemicals into the environment. Therefore, compounds with high inhibitory performance and low side effects have a high priority for biofouling control.

The phenomenon of biofouling is preceded by biofilm formation. Microbial adhesion corresponding to the first stages of biofilm formation (microfouling) is a critical step in biofouling. Although the initial adhesion of microfoulers in primary steps is reversible and achieved by physical forces such as Brownian motion, gravity, and water flow, following the maturation of the biofilm their presence becomes permanent (Cao et al., 2011). Formation of slime layers comprising surface-attached bacteria (mainly *Proteobacteria*) and their extracellular polymeric substances provide the prerequisite conditions for further attachment and increases the settlement of macrofouling organisms (Donlan and Costerton, 2002).

Detailed knowledge about biofilms is crucial for understanding and preventing biofouling. However, it is more practical to inhibit fouling formation at the initial phase. To discover non-toxic and eco-friendly benign anti-foulants, organisms such as typical free fouling organisms with natural chemical defense mechanisms have been investigated (Abarzua et al., 1999; Piazza et al., 2011; Dobretsov et al., 2013).

The most serious problem encountered in the development of derivatives of natural anti-fouling agents is producing large amounts of anti-fouling compounds using these organisms (Fusetani, 2011). To overcome this problem, other sources such as easily cultivable terrestrial and marine microorganisms including bacteria, fungi, and cyanobacteria have been explored in recent years (Qian et al., 2009). Among various habitats, marine environments provide great opportunity for discovery of novel bioactive compounds, due to intact, underexplored and highly bio-diverse ecosystems, compared to terrestrial environments.

The Persian Gulf’s marine environment is characterized by environmental extremes due to its location and bathymetry (Sale et al., 2011). The extreme salinity and temperature fluctuations of the Persian Gulf waters, which is one of the semi-enclosed marine systems, have created unique marine ecosystems (Bayani, 2016).

The phylum Actinobacteria is extraordinarily diverse and widely distributed in the marine environment (Maldonado et al., 2005; Pathom-Aree et al., 2006). Due to their large genome size and proven ability to produce structurally and functionally novel bioactive compounds, they hold a prominent position in biotechnological industries as sources of substitutes for commercial synthetic materials (Subramani and Aalbersberg, 2013). Therefore, marine Actinobacteria are a rich source of novel and biologically active products, with the potential to impede the adhesion of fouling organisms on artificial or natural surfaces. Herein, we investigated new marine Actinobacteria as a potential source of eco-friendly metabolites with anti-microfouling activity.

## Materials and Methods

### Isolation of the Marine Microfouler Bacteria

#### Experimental Field for Biofilm Formation on Artificial Surfaces

In order to isolate dominant fouling bacteria from the Oman sea, four different artificial substrates including wood, aluminum, steel and fiberglass were mounted on polyethylene holders and exposed to seawater at 4 m depth and left for 14 days at 31°C to form microfouling films. Each structure was collected after a 1-day interval and transferred to the laboratory under sterile conditions. Physicochemical parameters such as temperature, salinity, pH and dissolved oxygen of the water sample were also measured during the formation of films.

### Isolation of Initial Fouling Bacteria From Submerged Artificial Platforms

Each panel was washed with sterile artificial seawater to rinse the loosely attached cells, mud, and clay. Surface-attached bacteria (biofilm slime samples) were obtained through swabbing and scraping surfaces of the material, using sterile cotton swabs and razors. Media including Vaatanen nine salt solution (VNSS), Zobella marine agar (ZMA), and nutrient agar were used to cultivate microfouling bacteria at the early stage of the fouling process. Plates were incubated at 28°C for 5 days in the dark. The morphologically distinct biofilm bacterial colonies were purified and stored at 8°C for further analysis.

#### Assessment of Biofilm Formation by Crystal Violet Assay

The ability of the prevalent microfouling bacteria in the Oman sea to form static biofilms was determined in 96 well flat-bottom polystyrene microtiter plates (Biofil, China) as described (Stepanović et al., 2000). Each well of the microplates was filled with 300 μl of the test bacterial inoculums in nutrient broth supplemented with 1% glucose and 1% sucrose with a cell density of 0.2 at 600 nm, and incubated for 72 h at 37°C. Culture medium and planktonic cells, as well as loosely adhered bacteria, were removed by dual washing with sterile physiological saline. Firmly attached bacteria firstly were fixed with 250 μl methanol for 15 min, then stained with 250 μl crystal violet (0.05%, v/v). After 15 min, the stain was withdrawn and the wells were washed twice with sterile distilled water. Plates were air-dried at room temperature and the stain was ultimately solubilized by adding 250 μl of glacial acetic acid (33% v/v) for 15 min and its absorbance was recorded at 545 nm. Growth medium was included for sterility check. *Staphylococcus aureus* UTMC 1403 was used as positive control. Experiments were run in triplicate.

#### Selection of Optimum *in vitro* Condition for Biofilm Formation by Model Fouling Marine Bacteria

The impact of additive sugars in enhancing biofilm formation of two potent fouling bacteria was investigated by adding different concentrations (0.5 and 1% v/v) of three types of saccharide (glucose, sucrose, and fructose) alone and also in combined regimes. Then, glucose and sucrose 1% regimes as the biofilm formation enhancers were included in two different nutrient growth media of BHI broth (Merck, Germany) and nutrient broth (Merck, Germany) and the biofilm formation support of these two modified media were compared.

### Isolation of Marine Actinobacterial Strains

#### Sampling Sites and Sediment Samples Collection

The sediment samples were collected using a grab sampler from different Iranian sites of the Persian Gulf and the Oman sea, from a depth of 8–70 m between December 2013 and September 2014. Sediment samples were placed in sterile plastic bags and transported with ice pieces to the lab and were stored at 8°C for further studies.

#### Sample Preparation

Samples were heated at 50°C for 72 h to select heat-resistant spore-producing bacteria (including Actinobacteria) and decrease the colonization of unwanted fast-growing bacteria. Dried samples were then ground, sieved and transferred to sterilized falcon tubes for further analysis.

### Isolation Condition of Actinobacterial Strains

Serial dilutions of 10^-1^ to 10^-3^ of each treated sediment sample were homogenized in 3% natural sea salt solution and 200 μl of each sample was spread evenly on modified oligotrophic isolation media (Table 1). The isolation media were appended with Oman sea salt to select indigenous marine dwelling bacteria. Moreover, all media were prepared in 1/5 diluted form to simulate the oligotrophic conditions of the marine ecosystem. Isolation of colonies was conducted from the 14th to the 28th day of incubation at 28°C. Individual colonies observed on plates were subcultured on ISP2 agar medium (malt extract, 10 g; yeast extract, 4 g; glucose, 4 g; calcium carbonate, 2 g; agar 14 g in 1 L of distilled water, pH 7.5) until pure colonies were obtained. Ultimately, purified isolates were stored at -20°C in half strength ISP2 media containing 30% glycerol for further studies.

**Table 1:** The modified media used in isolation of marine-derived Actinobacteria from sediment samples.

Medium	Components	Reference
^∗^1/5 Glycerol asparagine agar (GA)	Glycerol, 2 g; asparagine, 0.5 g; K_2_HPO_4_, 1 g; NaCl, 0.5 g; MgSO_4_7H_2_ O, 0.5 g; FeSO_4_7H_2_O, 0.01 g; CuSO_4_, 0.001 g; MnSO_4_, 0.001 g; ZnSO_4_, 0.001 g; Agar, 14 g and 1 L of seawater, pH 7.5	Chen et al., 2012
1/5 Vaatanen Nine-Salt Solution (VNSS)	Glucose, 0.1 g; peptone, 0.2 g; yeast extract, 0.1 g; soluble starch, 1 g; FeSO_4_7H_2_O, 0.01 g; Na_2_HPO_4_ ⋅ 2H_2_O, 0.007 g; sea salt, 17.6 g; Na_2_SO_4_, 1.47 g; NaHCO_3_, 0.08 g; KBr, 0.04 g; KCl, 0.25 g; MgCl_2_ ⋅ 6H_2_O, 1.87 g; CaCl_2_ ⋅ 2H_2_O, 0.41 g; SrCl_2_ ⋅ 6H_2_O, 0.01 g; H_3_BO_3_, 0.01 g; agar, 14 g and 1 L of distilled water, pH 7.5	Heindl et al., 2012
1/5 “Reasoner’s 2A (R2A)	Yeast extract, 0.1 g; bacteriologic peptone, 0.1 g; casein hydrolysate, 0.1 g; glucose, 0.1 g; soluble starch, 0.1 g; sodium pyruvate, 0.06 g; agar, 14 g and 1 L of seawater, pH 7.2–7.4	Heindl et al., 2012
Modified HV agar (Natural sea water)	Fish powder, 0.1 g; CaCO_3_, 0.02 g; Na_2_HPO_4_, 0.5 g; MgSO_4_.7H_2_O, 0.5 g; KCl, 1.7 g; FeSO_4_.7H_2_O, 0.01 g; Vitamin solution, 1 ml; agar, 14 g; 1 L of sea water, pH 7–7.5	Sharma, 2014
	Vitamin solution consist of (biotin, 200 mg; pyridoxine HCl, 500 mg; thiamine HCl, 500 mg; riboflavin, 1 g; nicotinamide, 1 g; P-aminobenzoic acid, 100 mg 1 L of distilled water	
Modified HV agar (Soil extract)	Fish powder, 0.1 g; CaCO_3_, 0.02 g; Na_2_HPO_4_,0.5 g; MgSO_4_.7H_2_O, 0.5 g; KCl, 1.7 g; FeSO_4_.7H_2_O, 0.01 g; Vitamin solution, 1 ml; agar, 14 g; 1 L of soil extract, pH 7–7.5	Ramesh and Mathivanan, 2009
Modified Zobella marine agar	Peptic digest of animal tissue extract, 30 ml; yeast extract, 1 g; ferric citrate, 0.1 g; NaCl, 19.45 g; MgCl_2_, 8.8 g; Na_2_SO_4_, 3.240 g; sea salt, 1.8 g; KCl, 0.55 g; NaHCO_3_, 0.16 g; KBr, 0.08 g; SrCl_2_, 0.034 g; boric acid, 0.022 g; NH_4_NO_3_, 0.0016 g; Na_2_HPO_4_, 0.008 g; sodium silicate, 0.004 g; sodium fluorate, 0.0024 g; agar, 14 g; 1 L of distilled water, pH 7.2+_0.2	Satheesh et al., 2012


*^∗^All media are prepared in one-fifth diluted form.*

#### Fermentation and Extraction of Secondary Metabolites

Bacterial precultures were prepared by inoculating patches (1 cm × 1 cm) of well-grown colonies in 50 ml ISP2 broth culture medium, followed by incubation at 28°C with shaking at 160 rpm for 48 h. The fermentation medium was then inoculated with 5% of actively growing seed culture. After 7 days of incubation at 28°C, extraction was performed by shaking with the same ratio of ethyl acetate for 1 h, followed by supernatant separation using centrifugation at 4,000 rpm for 10 min. The organic phase was evaporated to dryness and concentrated using a rotary evaporator (Heidolph, Germany) at 35°C to obtain the bacterial extract.

### Anti-adhesion Activity in Presence of Crude Extract

To evaluate the inhibitory effect of the extracts on bacterial adhesion, 50 μl of each crude extract dissolved in methanol to a final concentration of 100 and 300 μg ml^-1^ was added to each well. 250 μl of fresh test bacterial cell suspension was then added to each well (final optical density of 0.2 at 600 nm) in nutrient broth media supplemented with 1% glucose and 1% sucrose, and the resulting mixtures were incubated at 37°C. After an optimal adherence time of 72 h, non-adherent bacteria in the spent medium were removed and the wells were washed twice with physiological saline. Fixation and staining steps were performed in the same manner using the crystal violet assay. The relative rate of adhesion to the bottom of wells in the presence of crude extract was calculated using the following equation (Bakkiyaraj and Karutha Pandian, 2010).

$$Adhesion(\%)=\frac{\left(\text{Control}\;\text{OD}-\text{Treated}\;\text{OD}\right)}{\text{Control OD}}\times 100$$

Microtiter wells containing bacterial cell suspension in nutrient broth medium without added extract were used as bacterial adhesion controls. In addition, the maximum percentage of methanol used for dilution of extracts and sterile nutrient broth medium was used as negative controls. Diuron (Sigma-Aldrich, United States), which is a commercially available anti-fouling agent, was used as a positive control (1 and 10 μg ml^-1^).

### Quantification of Extra Polymeric Substance (EPS) Content for Potent Biofilm-Forming Bacteria

Determination of EPS content of the selected marine fouling bacteria in the presence and absence of the extract at concentrations of 100 and 300 μg ml^-1^ was conducted by using the total carbohydrate assay, as follows. The culture broth from 72 h cultures of each microfouling bacterium was centrifuged at 14,000 × *g* and 4°C for 15 min to obtain the culture supernatant. The supernatant was passed through a 0.2 μm filter and the filtrate was mixed with ethanol at a ratio of 1:3, then left to precipitate at 4°C overnight. Settled EPS was collected by centrifugation at 10,000 × *g* and 25°C for 5 min, then dissolved in PBS and the concentrations of carbohydrates were determined with the modified phenol-sulphuric acid method using glucose as the standard (DuBois et al., 1956).

### Anti-bacterial Activities of the Anti-microfouling Extract Against the Marine Biofilm Forming Strains

The most effective extract with biofilm inhibition activity was evaluated for its inhibitory effect on marine biofilm forming bacteria using the agar disk diffusion method as described by Kirby-Bauer (Hudzicki, 2009). Firstly, 20 μl of the extract stock solution in 1 ml of methanol was applied on a sterile disk and allowed to dry such that the final concentrations on each disk were 5, 15, and 100 μg ml^-1^. The nutrient agar plates were seeded by spreading 100 μl of each test cell suspension (cell density of 0.2 at 600 nm), onto which the disks were placed and incubated at 35°C for 24 h. Standard antibiotic-containing disks [cefotaxime (30 μg), imipenem (10 μg), and ticarcillin (20 μg)] and filter paper disks soaked in methanol were used as positive and negative controls, respectively. Anti-microbial efficacy was determined by measuring the growth inhibition zone around the disks after 24 h.

### Bacteriostatic and Bactericidal Activity of Anti-microfouling Extracts

The minimum inhibition concentration value of the effective extract with the highest anti-adhesion and anti-bacterial activity was determined by the microdilution method following the CLSI procedure (Wayne, 2017). Briefly, 20 μl of each microfouling strain suspension with a cell density of 0.2 at 600 nm was added to each well containing extract at concentrations of 6.25–400 μg ml^-1^, along with an appropriate growth medium. Ciprofloxacin (0.25, 0.5, 1, 2, and 4 μg ml^-1^) was used as a positive control and well plates containing only bacterial suspensions were considered as the negative control. Following incubation at 35°C for 24 h, the lowest concentration at which the bacterial growth was inhibited and the one at which there was no visible bacterial growth on the agar plate was recorded as the MIC and MBC values, respectively.

### Quantification of Microfouler Metabolic Activity in Presence of Anti-microfouling Extract

The XTT [2,3-bis(2-methoxy-4-nitro-5-sulfophenyl)-2H-tetrazolium-5-carboxanilide] reduction assay was used to determine the bactericidal effects of the extract on single species biofilm populations as described (Padmavathi et al., 2014) with minor modifications. After biofilm formation of the tested bacteria in nutrient broth medium, the cell density was adjusted to 0.2 at 600 nm and combined with 50 μl extract solution to provide final concentrations of 50, 100, 200, and 300 μg ml^-1^ in a total volume of 300 μl per well. The wells were washed twice with 300 μl of sterile physiological saline. Then biofilms were incubated with 200 μl of XTT solution containing 150 mg XTT and 10 mg of phenazine methosulfate (Sigma-Aldrich) for 6 h in the dark, at 37°C at 120 rpm. The absorbance due to the formazan formed was measured at 490 nm using a MRP4+ microplate reader (Hyperion, England). Ciprofloxacin (2 and 4 μg ml^-1^) was used as a positive bactericidal reference. Wells containing plain culture medium without biofilms were used as blanks and biofilm containing wells without extracts were used as negative controls. The results were expressed as a percentage of inhibited activity.

### Eukaryotic Cell Toxicity Assay

Brine shrimp *Artemia salina* larvae were used as a model for detecting the toxicity of the extract. The anti-crustacean assay was performed in 24 well clear polystyrene plates (Meyer et al., 1982) in artificial seawater (3%) as the hatching solution. The extract was added at different concentrations (5, 10, 20 μg ml^-1^) and incubated at 25°C in the dark with 15–20 larvae in each well. Artificial seawater containing 1% DMSO and potassium dichromate solution (0.5 M), were used as the negative and positive controls, respectively. The Mortality rate was calculated after 24 h according to the following formula:

$$M=\frac{A-B-N}{G-N}\times N-0\;Viability=100\times \left(1-M\right)$$

Where *A* = dead larvae number after 24 h, *N* = dead larvae number initially, *M* = dead larvae percentage after 24 h, *G* = total number of larvae, and *B* = average number of dead larvae in negative control after 24 h.

### Hemolytic Activity Assay

The lytic activity of the extract was measured against human red blood cells. The red blood cells (10%) were separated by centrifugation at 3,500 rpm for 15 min and washed with PBS buffer three times. The most potent biofilm inhibiting extract was prepared at final concentrations of 50, 100, 200, 300, 400 μg ml^-1^ and added to the red cell suspension in PBS buffer. Mixtures of blood and extract were incubated at 37°C for 1 h. The supernatants were collected by centrifugation at 2,500 rpm for 5 min and optical densities were measured at 545 nm. Ferrous sulfate solution at a concentration of 65 mM was used as a positive control.

### Evaluation of Anti-microfouling Activity of the Actinobacterial Extracts in Field Experiments

The anti-adhesion activity of the extracts on two dominant fouling bacteria of the ecosystem was tested in field conditions following a modification of a literature method (Schwartz et al., 2017). Gels were prepared by adding 0.75 g of Gelrite^®^ (Dokhefa, Poland) to 50 ml of distilled water and the pH was adjusted to 7.1. The mixture was sterilized and then cooled to 70°C. Extracts were added to the liquid gel solution at a final concentration of 100 μg ml^-1^ and poured into sterile 48-well flat-bottom polystyrene microtiter plates. The same amount of methanol alone was added to Gelrite in the negative control well and Diuron (1 μg m^-1^) was used as a positive control. Bacterial strains were grown on nutrient broth supplemented with 1% glucose and 1% sucrose at 28°C and 120 rpm. The cells were collected once stationary phase had been reached. After centrifugation, cells were suspended in sterile artificial seawater at an optical density of 0.5 at 600 nm. Wells were seeded using 1 ml of fouling bacterial suspension and the resulting cultures were grown for 7 days at 32°C. Non-adherent bacteria were removed and the adhering bacteria were washed twice using physiological saline. The attached bacteria were moved from the gel surface into aqueous solution by sonicating at 40 KHz for 1 min using Elmasonic P sonicator (Elma, Germany). Finally, the adhesion rate of attached bacteria on the gel surface (*n* = 3) of each treatment and control were quantified spectrophotometrically at 600 nm.

### Characterization and Identification of Actinobacterium With Anti-microfouling Activity

The potent anti-adhesion producing strain was characterized using morphological, physiological, and molecular approaches.

#### Morphological Identification

Cultural characteristics based on observation of macro-morphology of the strain grown on ISP2 medium after incubation at 28°C for 14 days were recorded. Furthermore, the spore-bearing hypha and spore chains were directly examined using bacteria grown on ISP2 medium using the cover-slip technique and a Zeiss Merlin field emission scanning electron microscope (SEM), applying the SEM Smart software version 5.05.

#### Biochemical and Physiological Characteristics

Physiological characteristics, including decomposition of xenobiotics, enzyme activity, and utilization of different sole carbon and nitrogen sources were determined as described (Goodfellow et al., 2012; Stackebrandt, 2014). Growth at different temperatures (15, 28, 37, and 42°C), and pH values (4.0–12.0 at intervals of 2 pH units), and NaCl tolerance at concentrations ranging from 0 to 10% with intervals of 2.5% NaCl (w/v) were assessed after incubation at 28°C for 7–14 days on ISP2 medium. In addition, the chemotaxonomic marker diaminopimelic acid (DAP) in cell wall hydrolysates was analyzed using the thin layer chromatography (Staneck and Roberts, 1974).

### Molecular Identification of the Most Potent Anti-microfouling Actinobacteria and Selected Dominant Biofilm Forming Bacterial Strains

The 16S rRNA genes were amplified using a set of universal primers (Supplementary Table 1). Amplified chromosomal DNA obtained from PCR reactions was purified using a PCR purification kit (NucleoSpin^®^ Gel and PCR Clean-up). The 16S rRNA gene sequences were blasted against the EzBioCloud database (Yoon et al., 2017).

### Metabolic Profiling of the Most Potent Strain With Anti-microfouling Activity

The identification of specialized metabolites in the extract of *Glycomyces sediminimaris* UTMC 2460 was performed using a UHPLC-ESI-Q-TOF instrument with the following specifications: a Dionex UltiMate 3000 UHPLC connected to a Zorbax Eclipse Plus C18 column (100 × 2.1 mm, 1.8 μm) coupled to a Bruker MaXis IMPACT mass spectrometer. The mass spectrometer was operated in positive ion mode with a scan range of 50–3,000 *m/z*. The solvent system of water (A)-acetonitrile (B), each supplemented with 0.1% formic acid, was used for chromatography. A gradient of 5–100% B over 30 min was applied at a flow rate of 0.2 mL min^-1^. Calibration was performed with 1 mM sodium formate at the start of each run.

## Results

### Isolation of Marine Fouling Bacteria From Submerged Platforms in Oman Sea

Marine fouling bacteria of the region were isolated from biofilms or microfouling shaped on four types of immersed panels. A slime layer was visually observed on the submerged surfaces from 24 h and reached approximate 99% coverage in the second week of the immersion. Among various apparent colonies on three different nutritious media, four prevalent macro-morphological strain types were selected based on repetition on different platforms and successive detection on the isolation medium. Microscopic and macroscopic images of the prevalent initiators of the fouling phenomena in the Oman sea are shown in Table 2.

**Table 2:** Microscopic and macroscopic characteristic of the dominant marine biofilm bacteria obtained from Oman sea.

Strain	Macroscopic image	Microscopic image	Artificial surface types
*Psychrobacter* sp. UTMC 2516	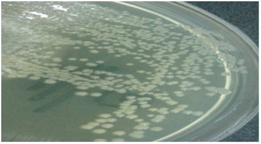	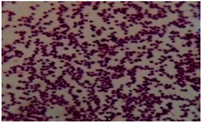	Woody panel
*Bacillus* sp. UTMC 2517	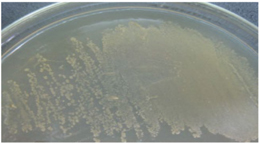	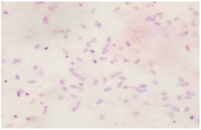	Steel panel
*Kocuria* sp. UTMC 2449	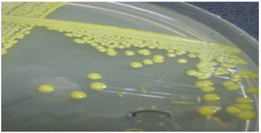	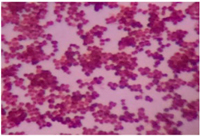	Fiberglass panel
*Mesorhizobium* sp. UTMC 2518	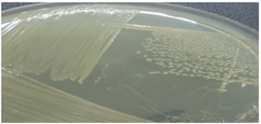	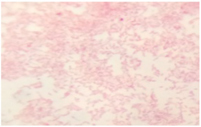	Aluminum panel


### Biofilm Forming Ability of the Selected Fouling Bacteria

**FIGURE 1:**
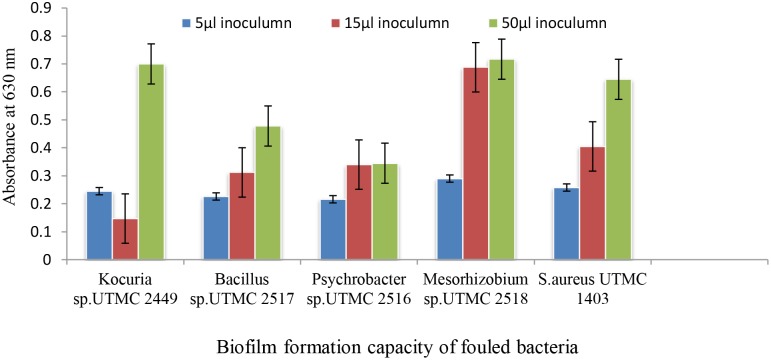
The biofilm formation of dominant isolated fouling bacteria from immersed artificial platforms in Oman sea detected by crystal violet assay.

The biofilm formation intensity of the isolated fouling strains was evaluated via the crystal-violet colorimetric assay. To compensate for background absorbance, the OD of the sterile medium with fixative and dye was recorded and subtracted from the results. The absorbance values of the crystal-violet served as an indicator of the total biomass of attached bacteria at the bottom of microplate wells. Bacterial strains didn’t show significant differentiation in biofilm formation ability at a low inoculum concentration (5 μl), while their ability to form biofilms varied at higher levels of inoculation (15 and 50 μl). Among four dominant fouling bacteria, two strains of *Kocuria* sp. UTMC 2449 and *Mesorhizobium* sp. UTMC 2518 showed the highest biofilm formation ability, which corresponded to an increase in optical density up to 0.7 at 600 nm (Figure 1). These two strains were selected as the primary fouling bacteria and used as the test microfoulers in further experiments. In this experiment *Staphylococcus aureus* UTMC 1403 was used as positive control strain due to its strong ability to form biofilms.

### Optimum *in vitro* Conditions for Biofilm Formation of Main Fouling Bacteria

The effects of growth medium supplementation with glucose, sucrose, and fructose were evaluated on the enhancement of biofilm formation by fouling bacteria. The results indicate that glucose and sucrose were more efficient sugars in comparison to fructose (Supplementary Figure 1), and the combination of glucose and sucrose 1% was more effective in the formation of the biofilm than each of them alone (Supplementary Figure 2). In addition, there was no significant difference between BHI and nutrient broth medium supplemented with 1% glucose and sucrose (Supplementary Figure 3). Hence, nutrient broth medium supplemented with 1% glucose and sucrose was selected as the medium supporting biofilm formation by *Kocuria* sp. UTMC 2449 and *Mesorhizobium* sp. UTMC 2518 for *in vitro* growth.

### Sediment Sample Collection and Isolation of Marine Actinobacteria

**FIGURE 2:**
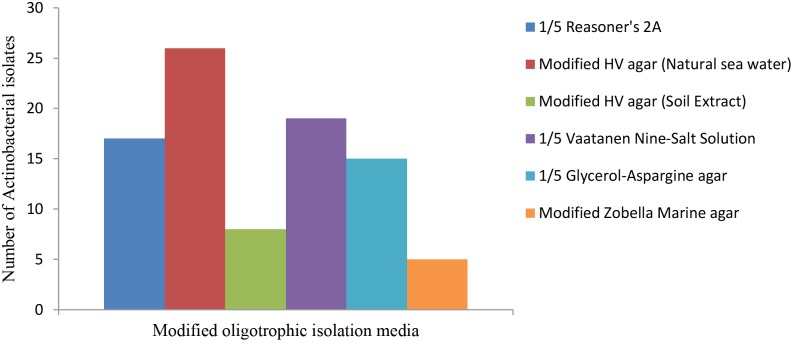
Comparison of the isolation media versus the number of recovered strains. Modified HV medium containing artificial seawater supported the highest number and diversity of the Actinobacteria.

A total of 50 sediment samples were collected from different sites between December 2013 and August 2014. The seawater physicochemical conditions at the test site were recorded. The average temperature, salinity level, pH and dissolved oxygen level at the sampling site were 32°C, 27.72 psu, 7.15 and 7.12 μmol l^-1^, respectively. A total of 90 strains were isolated from five oligotrophic isolation media. Modified HV medium containing artificial seawater provided the highest number and diversity of recovered strains with the minimum contaminants, followed by 1/5 Vaatanen Nine-Salt Solution and 1/5 R 2A (Figure 2).

### Production and Extraction of Metabolites From Marine Actinobacterial Strains

Among a total of 90 isolated actinobacterial strains from the sediment samples, 10 morphologically distinct isolates were selected for evaluation of the anti-microfouling activity of their metabolite extracts. Concentrated crude extracts were preserved in the University of Tehran bio-compound Collection.

### Anti-adhesion Activity of the Extracts

**FIGURE 3:**
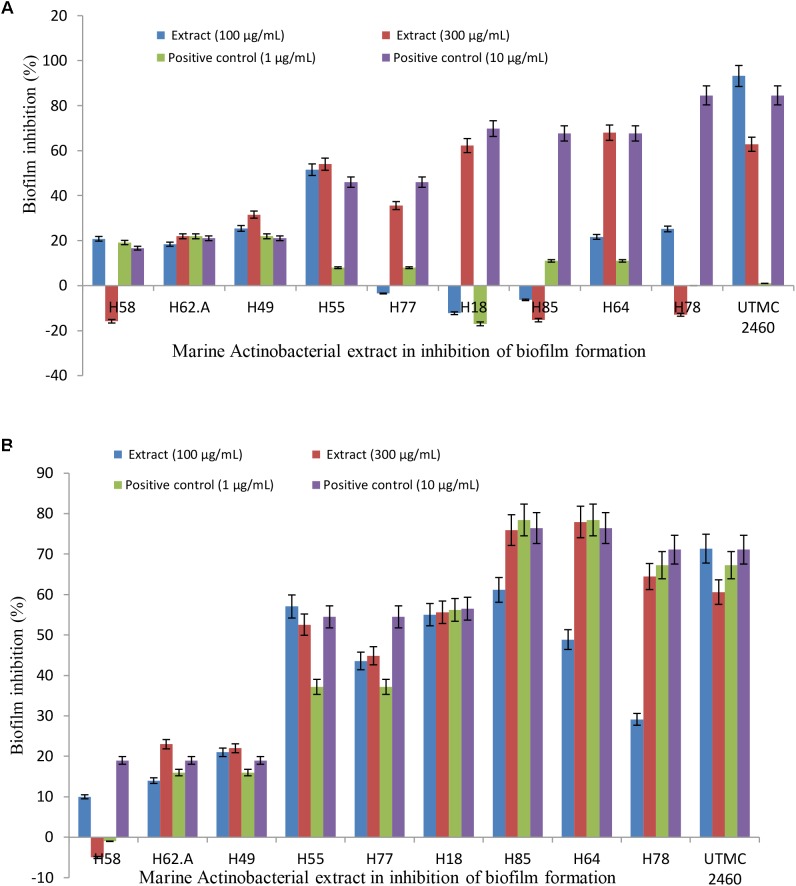
Anti-adherence activity of *Glycomyces* sp. UTMC 2460 metabolite against dominant marine fouled bacteria. The graph illustrating the potential of selected marine Actinobacterial extracts (100 and 300 μg ml^-1^) on the adhesion prevention of marine fouling bacteria: **(A)** *Kocuria* sp. UTMC 2449 and **(B)** *Mesorhizobium* sp. UTMC 2518 in crystal violet assay.

The ability of the selected marine actinobacterial extracts to attenuate biofilm formation by potent fouler bacteria from the Oman sea (*Kocuria* sp. and *Mesorhizobium* sp.) is shown in Figures 3A,B. The final results are expressed as the percentage of the reduction of adhered cells compared to a control that was not exposed to the extracts. Extracts with the highest anti-adhesion property at low concentration were selected for further analysis. All five initially selected extracts showed a concentration-dependent decrease in biofilm formation with the maximum biofilm inhibition activity at the lowest concentration value of 100 μg ml^-1^. The selected extracts caused 18–91% and 21–71% reduction in biofilm biomass production by *Kocuria* sp. UTMC 2449 and *Mesorhizobium* sp. UTMC 2518, respectively, at the minimum concentration of 100 μg ml^-1^. Most of the extracts exhibited stronger biofilm formation suppression against both fouling bacteria at the higher concentration of 300 μg ml^-1^, as shown in Figures 3A,B. Among five potent extracts, the extract from *Glycomyces* sp. UTMC 2460 showed the maximum anti-adherence activity against the fouling bacteria.

### Effect of Bacterial Extract on EPS Production of Microfouling Bacteria

**FIGURE 4:**
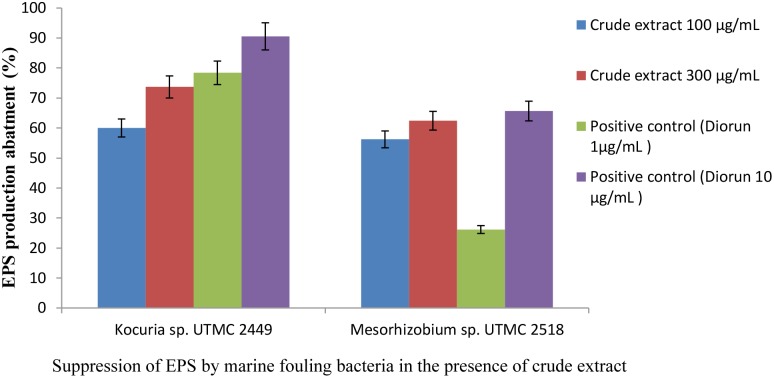
Inhibitory effect of *Glycomyces* sp. UTMC 2460 extract on EPS production of fouling bacteria. Respective samples incorporated with “Diorun” (1 and 10 μg ml^-1^) serve as controls.

A considerable decrease in EPS production by fouling bacteria in the presence of the extract from *Glycomyces* sp. UTMC 2460 was detected. The carbohydrate concentrations of the EPS extracted from *Kocuria* sp. UTMC 2449 suspensions were 92.19 μg ml^-1^ and 62.05 μg ml^-1^ in the presence of 100 and 300 μg ml^-1^ of the extract, respectively, while those in EPS isolated from *Mesorhizobium* sp. UTMC 2518 were 52.15 and 55.32 μg ml^-1^, respectively. The maximum reduction in EPS production (approximately 70%) was observed in *Kocuria* sp. UTMC 2449 in the presence of crude extract (Figure 4). The extract could diminish the EPS production in fouling bacteria (62–73%) with a similar efficiency of 1 and 10 μg ml^-1^ diuron on *Mesorhizobium* sp. and *Kocuria* sp., respectively.

### Antibacterial Effect of the Extract Against Main Fouling Bacteria

The antibacterial activity of the extract against the pelagic form of fouling bacteria was investigated due to its probable effect on the balance of the ecosystem. The zone of inhibition produced by the extract from *Glycomyces* sp. UTMC 2460 against *Kocuria* sp. UTMC 2449 at concentrations of 15 and 100 μg ml^-1^ was 3 mm and 8 mm, respectively (Supplementary Figure 4). No anti-bacterial activity for the extract was detected against *Mesorhizobium* sp. UTMC 2518 at concentrations up to 100 μg ml^-1^.

### Determination of the MIC and MBC Values of the *Glycomyces* sp. Extract

The minimal inhibitory concentration (MIC) and minimal bactericidal concentration (MBC) of the extract from *Glycomyces* sp. UTMC 2460 were determined for the fouler strains. The extract did not show growth inhibitory activity on fouling bacteria at concentrations less than 400 μg ml^-1^ and in fact the extract could not inhibit the fouler strains at low concentrations which has its anti-fouling effect. 100% bacteriostatic activity of the positive control against *Kocuria* sp. and *Mesorhizobium* sp. was exhibited at a concentration of 1 μg ml^-1^ for ciprofloxacin. The MBC value for ciprofloxacin was 2 μg ml^-1^. The *Glycomyces* sp. UTMC 2460 extract shows no bactericidal activity against two potent fouler bacteria in the planktonic phase.

### Bactericidal Effect of the Most Potent Extract Against the Fouling Bacteria in the Biofilm State

An XTT reagent incubation time of 6 h was found to be optimal since the color intensity did not change further after this period (data not shown). Evaluation of the viability and metabolic activity of *Kocuria* sp. UTMC 2449 and *Mesorhizobium* sp. UTMC 2518 in the presence of the *Glycomyces* sp. extract in the biofilm state revealed that *Kocuria* sp. UTMC 2449 was more susceptible to the extract at the concentration examined.

**FIGURE 5:**
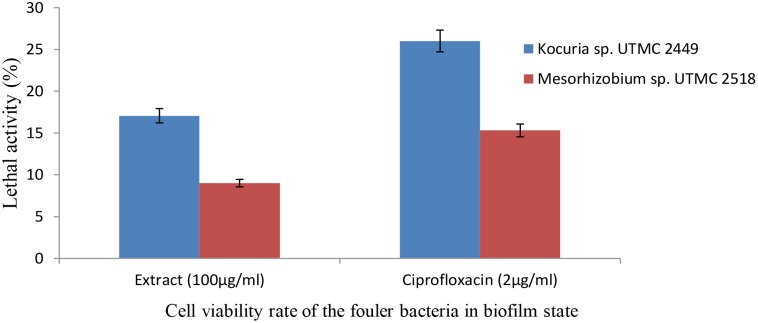
The effect of extract on the viability of *Kocuria* sp. UTMC 2449 and *Mesorhizobium* sp. UTMC 2518 cells in biofilm state. The lethal activity of the *Glycomyces* sp. UTMC 2460 metabolite at the lowest biofilm inhibition concentration (100 μg ml^-1^) was resembeled to ciprofloxacin as the control compound with bactericidal activity.

The metabolic activity at the minimum biofilm inhibition concentration of the extract (100 μg ml^-1^) reduced by up to 17% and 9%, respectively (Figure 5), after 6 h incubation with XTT reagent. The bactericidal effect of the extract was observed during the early phase of growth, with a 1.23 and 0.95 log reduction for *Kocuria* sp. UTMC 2449 and *Mesorhizobium* sp. UTMC 2518, respectively. Furthermore, *Kocuria* sp. UTMC 2449 and *Mesorhizobium* sp. UTMC 2518 biofilms subjected to the positive control presented 1.43 and 1.17 log metabolic reduction, respectively.

### Cytotoxicity Effect

The anti-crustacean assay provides an indication of the ecological safety of the anti-microfouling extracts. From the results obtained, it was inferred that all extracts expressing high anti-biofilm activity were not toxic toward *Artemia salina* larvae, because all exposed larvae were alive and motile after 24 h of incubation.

### Hemolytic Activity of the *Glycomyces* Extract

**FIGURE 6:**
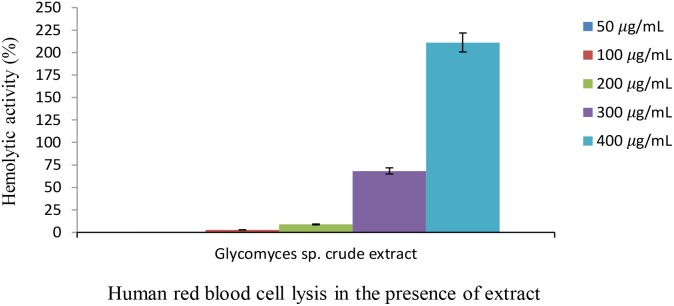
Hemolytic activity of *Glycomyces* sp. UTMC 2460 crude extract on human red blood cell. Almost no or a low hemolytic effect was detected at low concentrations of the extract (50, 100, and 200 μg ml^-1^). Hemolysis activity was evaluated in comparison to FeSO_4_ solution (65 mM) as a positive control.

The extract from the strain *Glycomyces* sp. UTMC 2460 had almost no or minimal (up to 8.8%) lytic effect at concentrations of 50, 100, and 200 μg ml^-1^ on human red blood cells (Figure 6). The biological safety of the most potent extract was inferred from its non-toxic effect on the blood cells. At the high concentrations of the extract (300 and 400 μg ml^-1^) toxicity was observed, presumably due to minor components of the extract.

### Anti-microfouling Activity of the Crude Extract in Simulated Field Conditions

**FIGURE 7:**
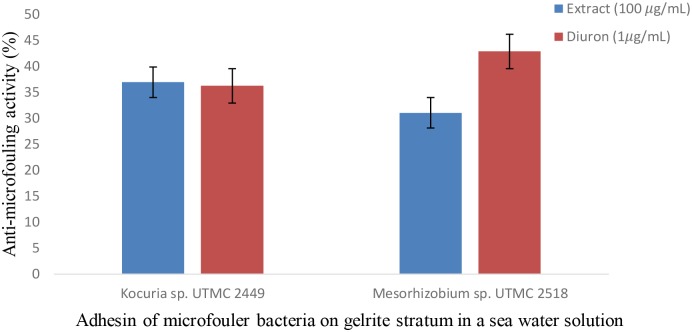
Anti-microfouling activity of *Glycomyces* sp. UTMC 2460 crude extract on two fouler bacteria in a simulated field condition. The extract exhibited 31% and 37% microfouling inhibition activity against *Mesorhizobium* sp. UTMC 2518 and *Kocuria* sp. UTMC 2449, respectively, in a mimicked field condition at *in vitro* experiment.

The effect of the *Glycomyces* extract on the adhesion of fouling bacteria was investigated using *in vitro* conditions that mimic the natural conditions of the Oman sea in terms of pH, salinity and temperature. The cell density of *Kocuria* sp. UTMC 2449 on the gels in the presence of the extract and in the positive control did not differ from each other and cell attachment was reduced up to 37%. The extract exhibited lower suppression of the adhesion of *Mesorhizobium* sp. UTMC 2518 (31%) than Diuron (1 μg ml^-1^). While the positive control showed a higher inhibitory effect on *Mesorhizobium* sp. UTMC 2518 (43%) than *Kocuria* sp. UTMC 2449 (36%) (Figure 7), the converse was observed for the extract.

### Molecular Identification of the Most Prevalent Marine Fouling Bacteria From Artificial Platforms

Results obtained from BLAST analyses of the 16S rRNA sequences from the fouling isolates against the GenBank and EzBioCloud databases showed that they belong to four genera, including *Psychrobacter*, *Kocuria*, *Bacillus* and *Mesorhizobium* (Table 3). These sequences were deposited in the GenBank database under the accession numbers shown in Table 3.

**Table 3:** Molecular identification of marine fouling bacteria based on 16S rRNA sequence.

UTMC			Accession
number	Strain name	Similarity (%)	number
2449	*Kocuria* sp.	*Kocuria atrinae* 99%	MH201002
2516	*Psychrobacter* sp.	*Psychrobacter faecalis* 95.5%	MH201003
2517	*Bacillus* sp.	*Bacillus atrophaeus* 99.8%	MH201004
2518	*Mesorhizobium* sp.	*Mesorhizobium amorphae* 98.5%	MH201005


### Polyphasic Identification of the Strain Producing the Anti-microfouling Activity

**FIGURE 8:**
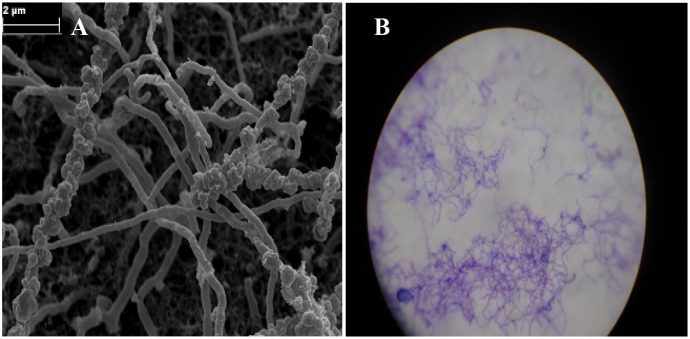
Micro-morphological characteristic of *Glycomyces sediminimaris* UTMC 2460 on ISP2 medium after 10 days’ incubation at 28°C. **(A)** Scanning electron micrograph of spore chains of the strain (Bar, 2 μm). **(B)** Filamentous vegetative mycelia under light microscopy.

Observation under a microscope indicated that the strain was Gram-positive with branching substrate mycelium and thus belongs to the phylum Actinobacteria (Figure 8B). Rod-like spores on aerial sporophores were observed under scanning electron microscopy (Figure 8A) and the strain formed small smooth colorless surface colonies on ISP2 medium. The optimum temperature and pH required for growth of the strain were 28°C and 7.5°C, respectively. The strain showed NaCl tolerance up to 5% (w/v). The physiological and biochemical characteristics of the strain are illustrated in Supplementary Table 2. The 16S rRNA was sequenced and BLAST analysis against the GenBank and EzBioCloud databases showed the highest similarity with *Glycomyces phytohabitant* (97.01%). The sequence of its 16S rRNA was deposited in the GenBank database under accession number KU1741966. The strain has been identified as a new species in the genus under the epithet of *Glycomyces sediminimaris* UTMC 2460, using a polyphasic assessment (Mohammadipanah et al., 2018).

### Characterization of the Metabolites in the *Glycomyces* Extract

**FIGURE 9:**
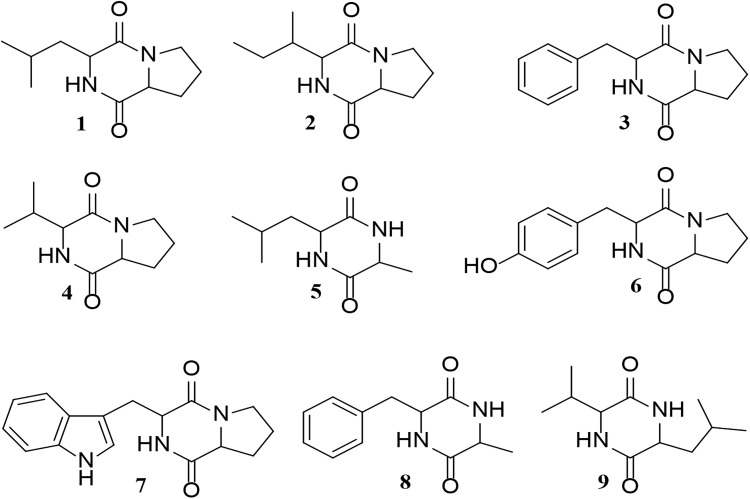
Structure of the putative diketopiperazines identified in the extract of *Glycomyces sediminimaris* UTMC 2460: 2,5-diketopiperazines cyclo-(leucyl-prolyl) (**1**), cyclo-(isoleucyl-prolyl) (**2**), cyclo-(phenylalanyl-prolyl) (**3**), cyclo-(prolyl-valyl) (**4**), cyclo-(alanyl-leucyl) (**5**), cyclo-(prolyl-tyrosyl) (**6**), and cyclo-(prolyl-tryptophyl) (**7**), cyclo-(alanyl-phenylalanyl) (**8**), and cyclo-(leucyl-valyl) (**9**).

UHPLC-ESI-Q-TOF-MS analyses indicated that the majority of the metabolites in the *Glycomyces sediminimaris* UTMC 2460 extract have molecular formulae and retention times corresponding to the diketopiperazine class of natural products (Supplementary Table 3 and Supplementary Figure 5). The major constituents are proposed to be cyclo-(leucyl-prolyl) (**1**), cyclo-(isoleucyl-prolyl) (**2**), cyclo-(phenylalanyl-prolyl) (**3**), cyclo-(prolyl-valyl) (**4**), cyclo-(alanyl-leucyl) (**5**), cyclo-(prolyl-tyrosyl) (**6**), and cyclo-(prolyl-tryptophyl) (**7**), cyclo-(alanyl-phenylalanyl) (**8**), and cyclo-(leucyl-valyl) (**9**) (Figure 9).

## Discussion

The establishment of micro- and macro-organism communities either on living or non-living substrate (biofouling) generally involve a sequence of succession started by the colonization of surfaces by bacterial biofilm formation (Wahl, 1989) and macrofouler settlement is controlled by prevention of biofilm formation (Salta et al., 2013). Therefore, bacterial adhesion is probably one of the most critical steps to target in the search for an efficient antifouling. Although, the majority of commonly used anti-fouling paints are based on biocidal agents that induce general toxic responses in the marine environment associated with heavy metal toxicity and antibiotic toxicity, some natural anti-fouling compounds from diverse source have been identified (Fusetani, 2011). However, bacterial sources are preferred as their reproducible process of cultivation and scale up that ensure product supply for commercialization (Dobretsov et al., 2006; Gademann, 2007; Qian et al., 2007). Actinobacteria pose a dominant status in the production of novel bioactive compounds including antifouling agents such as lobocompactol (Cho and Kim, 2012), quercetin (Gopikrishnan et al., 2016) and diketopiperazines (Li et al., 2006).

The majority of Iran marine ecosystems are poorly explored. Until the beginning of 2018, less than 1% of previous studies in Iran marine habit was conducted on marine bacteria and fungi and several less known/unknown taxa are still waiting to be identified (Maghsoudlou et al., 2017). For this reason, Actinobacterial strains were isolated from subtidal sediment samples of the Iranian waters in the Persian Gulf and the Gulf of Oman and their anti-adhesion activities was evaluated versus commercial and natural anti-fouling products against dominant microfouling initiator of this ecosystem.

The investigation of the structure of pioneer fouling communities on four kinds of submerged artificial substrate (wood, fiberglass, aluminum, and steel) in Oman Sea revealed different dominant species compared to the other ecosystems. Unlike other previous studies which illustrated γ-Proteobacteria such as *Pseudoalteromonas*, *Shewanella*, and *Vibrio* (Lee et al., 2003) predominant in marine biofilms, in this study the prevalent initial marine fouling bacteria belonged to *Mesorhizobium, Kocuri*a, *Bacillus* and *Psychrobacter* genera in which *Mesorhizobium* sp. *and Kocuri*a sp. formed strict biofilm structures in *in vitro* conditions and were selected as the potent microfouler strains in the complementary analysis. The composition of bacterial biofilm communities during the first stage of biofouling period, not only differs based on the seawater in which the substrata of the biofilms were submerged but also depends on the time course and surface type. Despite the isolation of *Kocuria* members from different marine sources Dastager et al. (2014) and Pote and Bhadekar (2014), there hasn’t been any reports demonstrating the biofilm forming abilities of this genus rather than their degradation activity. Hence, this result can trigger further studies in the field of biofilm formation and quorum sensing investigations of this genus. Based on different reports, (Prasad et al., 2014; Yong and Chan, 2014), *Mesorhizobium* species have been isolated from seawater and natural surfaces which were immersed in the aqua environment. In addition, they found that *Mesorhizobium* species have the quorum sensing abilities; hence our reported data about the strict biofilm formation ability of the isolated *Mesorhizobium* sp. UTMC 2518 from an aluminum platform is in line with the previous report on this genus (Yong and Chan, 2014).

In this research, the criteria for selection of artificial substrates (fiberglass, steel wood, and aluminum) were based on the frequency of usage and application of materials in the marine infrastructure equipment in Persian Gulf ports provenance of Iran. As mentioned, the surface type of immersed panels in which biofilms formed on has a fundamental role in the composition and accumulation rate of bacterial communities in biofilms sampled during the first stage of biofouling. The observed data in field experiments and former studies demonstrate that surface roughness aids the physical retention of cells by enhancing adhesive contact, protecting from hydrodynamic forces, grazing activity and desiccation (Fletcher and Callow, 1992), and settling on hydrophobic compared to hydrophilic self-assembled monolayers in static assays is higher (Callow et al., 2000). The highest distribution rates of fouling population on different experimental panels were in the order of wood > fiberglass > steel > aluminum which was in line with the settled diversity. Low fouling rate on steel and aluminum panels can be originated from hydrophilic properties of the panel or viable toxicity of metal surface. As a result of this, due to high amounts of fouling on the second week immersed panels, isolation of primary marine fouling bacteria from these panels was replaced by 24-h, and 72-h immersed panels were considered. Despite higher fouling rates on wood panels, a variety of initial marine biofilm forming bacteria were obtained in the following isolation order fiberglass > steel > aluminum > wood.

In the second part of this study, in the Actinobacterial strains isolation process, modifications were applied to the isolation media to simulate oligotrophic conditions. Amongst the retrieved Actinobacteria, approximately 30% were separated from modified HV medium (containing NSW) which was the most oligotrophic in comparison with other media. In respected studies, 12 GYA medium and HV-agar medium were used, respectively, for isolation of Actinobacteria from sediment and seawater samples, demonstrating the effectiveness of oligotrophic conditions in strain isolation (Gebreyohannes et al., 2013; Sharma, 2014).

Deterrence of marine fouling is linked to the control of attachment and development of microfouling communities. The inhibition of adhesion may result from two mechanisms, i.e., a specific inhibition of the adhesion process or a consequence of toxicity toward the strain(s). In the present study, the anti-microfouling activity of the ten marine Actinobacteria from deep-sea sediments of the Persian Gulf was screened through anti-attachment assay, of which the crude extract of five strains demonstrated distinct anti-adherence activity against the settlements of potent biofilm-forming bacteria. The extract from *Glycomyces sediminimaris* UTMC 2460 strain exhibited the adherence inhibition in range of 72–95% with no or negligible antibacterial effect on two potent fouler bacteria in the planktonic state. This indicated limited bacterial toxicity and growth inhibition activity of the extract on the dominant microflora of this ecosystem. In accordance with the present study, the anti-microfouling activity of the crude extract of *Streptomyces filamentosus* R1 and *S. fradiae* against the marine fouling bacterial strains, such as *Bacillus* sp., *Serratia* sp., and *Alteromonas* sp. is reported by Bavya et al. (2011) and Prakash et al. (2015), respectively. Despite the lower antibacterial effect of *G. sediminimaris* UTMC 2460 extract on the planktonic form of the microfoulers in comparison to similar aforementioned studies, its mechanism of action has the privilege to be delivered by the biofilm formation inhibition. The effect of any anti-fouling agent on the proliferation of adhered strains has the advantage of effect on planktonic cells as they will not harm the balance of the microflora in the marine habitats.

Extra polymeric substance as a crucial and integral part of a biofilm plays a pivotal role in the durability of a biofilm through the adhesion and aggregation of other microfoulers. Therefore, abating EPS production can arrest the biofilm formation which holds promise for the development of functional anti-fouling agents. It can be concluded that, the high potent anti-adherence activity of Actinobacterial extract against *Kocuria* sp. UTMC 2449 and *Mesorhizobium* sp. UTMC 2518 biofilm can also be attributed to the considerable inhibition in EPS production.

Besides anti-microfouling efficacy, toxicity is a major concern for marine coating industry as effective marine natural compounds are sometimes as toxic as heavy metals. Thus, the toxicity profiles of the five potent anti-microfouling extracts were also determined in this study. The absence of mortality effect on the larvae of *Artemia salina* mitigating their ecological concern. Additionally, compared with the LC_50_ of tributyltin, which is generally less than 0.00001 μg ml^-1^, (Cardwell et al., 1999) these results indicate that these extracts are much less toxic than tributyltin. The extract of *Glycomyces sediminimaris* UTMC 2460 as the most effective extract on fouling inhibition of *Kocuria* sp. UTMC 2449 and *Mesorhizobium* sp. UTMC 2518, exhibit not only no toxic effect on *Artemia salina* but also has a negligible hemolytic activity which is an indication of acute toxicity effect. Therefore, it can be concluded that the crude extract of *Glycomyces sediminimaris* UTMC 2460 can be considered as an environmentally safe anti-fouling agent.

Analysis of the crude extract of from *Glycomyces sediminimaris* UTMC 2460 identified nine compounds corresponding to the family of diketopiperazines. Diketopiperazines are known to possess potent anti-fouling activity (Li et al., 2006; Cho et al., 2012) and the anti-fouling properties of seven of the nine diketopiperazines tentatively identified in this study have already been investigated. These include compounds **1**, **3**, **4**, **7**, and **9** (Li et al., 2006), and metabolites **5** and **8** (Cho et al., 2012). The anti-fouling activity of *Glycomyces sediminimaris* UTMC 2460 is therefore most likely attributed to the production of diketopiperazines **1–9**.

## Conclusion

The overall results of the present study clearly emphasized the strain *Glycomyces sediminimaris* UTMC 2460 as a competent producer of the anti-microfouling natural product and substantiated its low toxic nature. Furthermore, the physiological, molecular and chemotaxonomical analysis, allowed its introduction as a new species in *Glycomyces* genus called *Glycomyces sediminimaris*. The sufficient studies to explore the bioactivity of the *Glycomyces* strains have not been performed and only a single report exists on the anti-tumor and antibiotic activity of *G. harbinensis* secondary metabolite (Lee et al., 1986). Hence, this report adds new valuable biological activity to this genus capability and can accelerate the investigation of other novel bioactivities from this genus and the species of *G. sediminimaris*. Further work on the anti-microfouling activity of the putative diketopiperazines produced by *G. sediminimaris* and field trial experiments are demanding to development a novel eco-friendly compound that is introduced as a potential substitute for commercial chemical biocide in the maritime industry.

## Author Contributions

SH performed the experiments and analysis and wrote the draft of the manuscript. FM and AM contributed as supervisors. FM defined the approach, designed the experiments, and edited the manuscript content. AM managed the water/sediment sampling. YD and GC performed the metabolite profiling, wrote the related sections, and contributed to the editing of the manuscript. All authors read and approved the final version of the manuscript.

## Conflict of Interest Statement

The authors declare that the research was conducted in the absence of any commercial or financial relationships that could be construed as a potential conflict of interest.
