# Supplementary material for: Anti-microfouling Activity of Glycomyces sediminimaris UTMC 2460 on Dominant Fouling Bacteria of Iran Marine Habitats
Source: Front Microbiol. 2019 Jan 9;9:3148. doi: 10.3389/fmicb.2018.03148 (PMC6333643; doi:10.3389/fmicb.2018.03148)
Supplement: Supplementary file 1 [file Data_Sheet_1.DOCX]

Supplementary Material

**Anti-microfouling activity of *Glycomyces sediminimaris* on dominant fouling bacteria of Iran marine habitats**

**Sheida Heidarian^1^, Fatemeh Mohammadipanah^1*^, Abdolvahab Maghsoudlou^2*^, Yousef Dashti^3^, Gregory L. Challis^3,4,5^**

**Co-Correspondence:** Fatemeh Mohammadipanah, [fmohammadipanah@ut.ac.ir](mailto:fmohammadipanah@ut.ac.ir) and Abdolvahab Maghsoudlou, [wahab@inio.ac.ir](mailto:wahab@inio.ac.ir)

**Supplementary Table 1.** The sequence of used primers in 16S rRNA gene amplification of the strains

| **Primer sequence (5'→3')** | **Primer name** |
| --- | --- |
| AGAGTT TGATCCTGGCTCAG | 27F |
| GTATTCCGCGGCTGCTGG | 518R |
| CTCCTACGGGAGGCAGCAG | 357F |
| AGGGTTGCGCTCGTTG | 1100R |
| YAACGAGCGCAACCC | 1100F |
| AAGGAGGTGWTCCARCC | 1525R |

**Supplementary Table 2.** Biochemical and physiological characterization of the selected potent marine strain, *Glycomyces* sp. UTMC 2460. +, positive; -, negative

| **Physiological characteristic** | | | | | | | | | | | | | | |
| --- | --- | --- | --- | --- | --- | --- | --- | --- | --- | --- | --- | --- | --- | --- |
| **Growth at:** | | | | | | | | | | | | | | |
| Temperature Range | | | 28- 37 | | | | Growth at 10^o^C | | | | | - | | |
| NaCl Range | | | 0-5 % | | | | Growth on 7% NaCl | | | | | - | | |
| pH ranges | | | 6-11 | | | |  | | | | | | | |
| **Sole carbon consumption:** | | | | | | | | | | | | | | |
| D- Fructose | + | Cellulose | | | + | Maltose | | | | + | Sodium citrate | | | + |
| Inositol | + | Cellobiose | | | + | D-Rhamnose | | | | + | Sodium acetate | | | + |
| D-Lactose | + | Galactose | | | + | Oxalate | | | | + | Sodium propionate | | | + |
| **Sole nitrogen consumption:** | | | | | | | | | | | | | | |
| L-Arginine | | | | L-Histidine | | | | | L-Cysteine | | | | L-Tyrosine | |
| + | | | | + | | | | | + | | | | + | |
| **Hydrolysis of:** | | | | | | | | | | | | | | |
| Casien | | | | Aesculin | | | | Starch | | | | | Nitrate reduction | |
| + | | | | + | | | | - | | | | | + | |

**Supplementary Table 3.** The dereplication results of the extract of *Glycomyces sediminimaris*

| m/z | Rt (min) | Molecular formula | Compound |
| --- | --- | --- | --- |
| 261.1232 | 12.3 | C_14_H_16_N_2_O_3_ | 6 |
| 197.1286 | 12.4 | C_10_H_16_N_2_O_2_ | 4 |
| 219.1131 | 13.0 | C_12_H_14_N_2_O_2_ | 8 |
| 211.1444 | 13.9 | C_11_H_18_N_2_O_2_ | 1 |
| 211.1445 | 14.2 | C_11_H_18_N_2_O_2_ | 2 |
| 245.1289 | 14.6 | C_14_H_16_N_2_O_2_ | 3 |
| 284.1395 | 14.8 | C_16_H_17_N_3_O_2_ | 7 |
| 185.1288 | 15.0 | C_9_H_16_N_2_O_2_ | 5 |
| 213.1600 | 16.8 | C_11_H_20_N_2_O_2_ | 9 |

**Supplementary Figure 1.** Evaluation of sugar supplements (Glucose, Fructose and Sucrose) on the improvement of biofilm formation of *Kocuria* sp. UTMC 2449 and *Mesorhizobium* sp. UTMC 2518. Two sugars (glucose and sucrose) showed a profound impact on the enhancement of *in vitro* biofilm formation by fouled bacteria

**Supplementary Figure 2.** The effect of the selected additives) glucose and sucrose) complemented to growth medium (nutrient broth) in enhancing the *in vitro* biofilm formation of *Kocuria* sp. UTMC 2449 and *Mesorhizobium* sp. UTMC 2518. Combination of two sugars was rather efficient in promoting biofilm formation of the fouled bacteria. Growth medium without bacterial inoculum served as blank

**Supplementary Figure 3.** Investigating the impact of two different nutrient media on the *in vitro* biofilm formation of *Kocuria* sp. UTMC 2449 and *Mesorhizobium* sp. UTMC 2518. Two different amounts of bacterial inoculums (15 and 50 𝜇l) with a cell density of 0.2 at OD 600 nm were used in the experiment

| 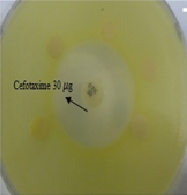  **A** | 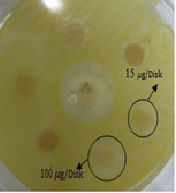  **B** |
| --- | --- |

**Supplementary Figure 4.** The low anti-microbial activity of *Glycomyces* sp. UTMC 2460 extract on the non-biofilm culture of fouler bacteria of *Kocuria* sp. UTMC 2449 and *Mesorhizobium* sp. UTMC 2518. **(A)** Growth inhibition zone diameter at the concentration of 15 and 100 𝜇g/disk was 3 mm and 8 mm, respectively; and **(B)** was 30 mm for the positive control (Cefotaxime 30 𝜇g/disk)


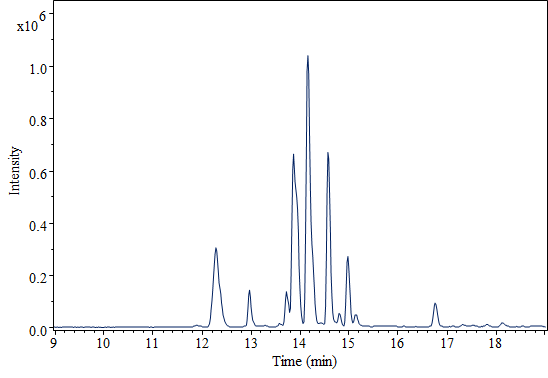


**Supplementary Figure 5.** UHPLC-ESI-Q-TOF-MS spectrum of *Glycomyces sediminimaris* extract.
